# Infant-Feeding Practices Among Women Living With Human Immunodeficiency Virus (HIV) in a Southern Nigerian Region: A Mixed Comparative Study

**DOI:** 10.7759/cureus.35483

**Published:** 2023-02-26

**Authors:** Ubong Akpan, Ezukwa Omoronyia, Kazeem Arogundade, Udeme Asibong, Adaolisa Nwagbata, Chinyere Akpanika, Saturday Etuk

**Affiliations:** 1 Department of Obstetrics and Gynaecology, University of Calabar Teaching Hospital, Calabar, NGA; 2 Department of Public Health, Bruyere Research Institute, Ottawa, CAN; 3 Department of Family Medicine, University of Calabar Teaching Hospital, Calabar, NGA

**Keywords:** wlwha, infant feeding, exclusive replacement feeding, mixed feeding, mother to child transmission of hiv, exclusive breastfeeding

## Abstract

Background and objective

Infant survival depends on proper nutrition. Breastfeeding enhances infant health and offers some benefits to the mother as well. However, in the setting of the HIV pandemic, it is important to assess the benefits and the risk for each individual in choosing a feeding option. The purpose of this research was to determine the infant-feeding practices among women living with HIV/AIDS (WLWHA) and compare them with the general population of women.

Methods

A mixed comparative survey was conducted among 246 HIV-positive mothers nursing infants of at least one year of age. An equal number of matched HIV-negative women from the same locality were selected as controls. Quantitative data were analyzed using IBM SPSS Statistics version 23 (IBM Corp., Armonk, NY) while qualitative findings were presented in a thematic approach.

Results

The prevalence of exclusive breastfeeding (EBF) among WLWHA was 73.6% compared to 55.2% in the controls [p=0.002; chi-square (X^2^)=5.264]. Only 6.5% of WLWHA practiced exclusive replacement feeding (ERF). Vaginal birth was associated with increased odds for early initiation of breastfeeding [p=0.001; odds ratio (OR): 3.135; 95% confidence interval (CI): 2.130 to 4.616]. Also, urban dwellers commenced breastfeeding earlier than women residing in rural communities (p=0.002; OR: 5.58; 95% CI: 3.85 to 8.07). Based on in-depth interviews, cultural influences and non-disclosure of HIV status to family members promoted mixed feeding. Concomitant intake of anti-tuberculosis drugs was a major reason for adopting ERF in some women.

Conclusion

There was a high prevalence of EBF among WLWHA. Counseling on infant feeding is an effective component of the Prevention of Mother-to-Child Transmission (PMTCT) programs. Cultural beliefs and fear of stigmatization are major challenges to infant nutrition in sero-exposed babies.

## Introduction

Breastfeeding is a unique source of nutrition for newborn mammals, including humans, and is necessary to enhance infants’ survival [1,2]. However, many blood-borne infections are also transmitted through breast milk. In the last three decades, the HIV pandemic has become a major threat to infant survival and breastfeeding has been identified as a major risk factor for vertical transmission of HIV during the postnatal period [3-5].

Appropriate infant feeding is therefore a persistent challenge for HIV-infected mothers. In resource-poor settings, it is difficult for infected mothers to meet the criteria of exclusive replacement feeding (ERF) with breast milk substitutes such as cow milk and other infant formulae [6,7]. These criteria, often abbreviated as AFASS, imply acceptability, feasibility, affordability, sustainability, and safety. The practices are often dependent on the socioeconomic factors of the individual or her family and may also be influenced by traditional or societal beliefs and norms [8,9].

Recent studies suggest that exclusive breastfeeding (EBF) is low in the general population of women globally, ranging from 20 to 60% [9-14]. A recent National Demographic and Health Survey (NDHS) reported that about 70% of Nigerian women practiced mixed feeding in the first six months of their infants’ lives [15]. Mixed feeding may enhance HIV transmission because the potential inflammation of the delicate digestive system of the infant induced by the breast milk substitute may allow for easy absorption of the virus from breast milk in the infant’s intestines [16]. EBF for the first six months is therefore associated with a significantly lower risk of HIV transmission as compared with mixed feeding [17-19].

For the past two decades, about 95% of infants delivered by HIV-positive mothers have been prevented from contracting HIV infection through the implementation of the Prevention of Mother-To-Child Transmission (PMTCT) intervention programs including safe infant-feeding practices [13,20,21]. This evidence has led to the inclusion of infant-feeding practices as a major component of antenatal HIV counseling and testing [22,23]. However, no recent research has been conducted in this southern Nigerian region to document the actual infant-feeding practices and assess the effectiveness of such antenatal Care (ANC) interventions among HIV-positive mothers despite the region having the highest HIV prevalence in Nigeria.

## Materials and methods

Research setting

The research was conducted in the Old Calabar region of Nigeria, comprising the present Calabar South, Calabar Municipality, Akpabuyo, and Bakassi local government areas. The region has two tertiary hospitals, two government-owned secondary health facilities, and more than 20 primary health centers where PMTCT and President Emergency Plan for Aids Relief (PEPFAR) programs are executed. With a population of about 650,000 and an HIV prevalence of 1.7% [24], there were about 11,000 people living with HIV in this region during the period of the survey and about 65% of them were females. The majority of the residents in the region are middle- and low-income earners (<300 US Dollars per month) depending on the prevailing exchange rates.

Research population

The research population comprised HIV-positive women of reproductive age who had nursed infants of at least one year of age. Equal numbers of HIV-negative women of similar age and parity brackets were recruited from postnatal clinics, general outpatient wellness clinics, and family planning clinics in the various health facilities as controls.

Sample size determination

The sample size for prevalence studies was calculated based on the NDHS of 2018, which reported a 29% prevalence of EBF among the general population. A total of 500 respondents were selected for the study group (n=250) and the controls (n=250).

Sampling method

A multistage sampling approach was adopted. In the first stage, one of the two tertiary health institutions - the University of Calabar Teaching Hospital - and a secondary health facility, General Hospital Calabar were selected based on the patient load data from PMTCT records. Then a list of all the primary healthcare (PHC) facilities in the region was obtained from the State Ministry of Health. Ten PHCs were then selected by lottery method. At the facility level, participants were selected purposively and using the snowball method until the required sample size was attained. At the PMTCT and PEPFAR units of the facilities, Women Living With HIV/AIDS (WLWHA) were surveyed while HIV-negative mothers who were attending maternity and child health clinic, immunization clinics as well as family planning clinics were selected in the same facilities as the control group. Women who declined consent were excluded. Twenty-five WLWHA, including three who practiced ERF, were randomly selected for in-depth interviews. Participants were encouraged to freely express their thoughts and concerns.

Ten health workers including three doctors, five nurses, and 2 community health officers were trained as research assistants. The instrument of quantitative data collection was a semi-structured interview administered via a questionnaire. Data on social demographic profiles, obstetrics and medical information, and specific information on infant-feeding practices were elicited using the questionnaire. The recorded information from the in-depth discussion was summarized by the lead researcher. The study was conducted between July 1, 2019, and December 2019.

Ethical issues

Approval was obtained from the Cross River State Ministry of Health Research Ethics Committee before the commencement of the study (approval no: CRS/MH/HREC/019/Vol.V1/168). Participation in the research was voluntary and full confidentiality was ensured. The names and identities of the participants were kept confidential.

Data analysis

The data were entered into the IBM SPSS Statistics version 23 (IBM Corp., Armonk, NY) for analysis. Descriptive data were presented in simple proportions and percentages while inferential statistics were used to assess relationships among determinant factors. The student chi-square test was used to assess the relationship between categorical variables. Logistic regression was computed to assess the relationship between dependent and independent variables. The level of significance was set at p≤0.05. Information from the in-depth discussion was presented using a thematic approach.

## Results

A total of 492 completed questionnaires, 246 from the study arm and the same number from the control group, were included in the final analysis. Their demographic characteristics were similar apart from educational status (Table 1). All WLWHA were on triple anti-retroviral (ARV) drugs for the treatment of HIV. The medications were initiated before or during the index pregnancy (ANC period) except in four (1.85%) who commenced the medication during the postnatal period. Their babies also received ARV prophylaxis.

**Table 1:** Sociodemographic profile of the respondents *Statistically significant CI: confidence interval; OR: odds ratio; TBA: traditional birth attendant

Variables		Study arm	Control arm	P-value	OR	95% CI	
		N=246	N=246				
		N (%)	N (%)			Lower	Upper
Age	<30 years	134 (54.5)	130 (52.8)	0.092	1.068	0.749	1.522
	≥30 years	112 (45.5)	116 (47.2)				
Marital status	Married	228 (92.7)	236 (95.9)	0.120	0.537	0.234	1.188
	Unmarried	18 (7.3)	10 (4.1)				
Parity	Primiparity	137 (55.7)	102 (41.5)	0.529	1.123	0.786	1.606
	Multiparity	109 (44.3)	144 (58.5)				
Income	Less than $300/month	169 (68.7)	172 (69.9)	0.717	0.932	0.634	1.368
	≥$300/month	77 (31.3)	74 (30.1)				
Domestic staff	Yes	175 (71.1)	188 (76.4)	0.153	0.760	0.508	1.138
	No	71 (28.9)	58 (23.6)				
Place of last childbirth	Health facility	212 (86.2)	210 (85.4)	0.796	1.059	0.844	1.773
	Home/TBA	34 (13.8)	36 (14.6)				
Nature of apartment	Single room	112 (45.5)	96 (39.0)	0.121	1.350	1.933	0.943
	Flat/duplex	134 (54.5)	150 (61.0)				
Education	Primary or lower	151 (61.4)	49 (19.9)	0.001*	4.88	3.24	7.86
	Secondary or tertiary	95 (38.6)	197 (80.1)				

Among WLWHA, a total of 181 out of the 246 respondents practiced EBF while 137 of the controls practiced EBF. Thus, the prevalence of EBF was higher among the study group (WLWHA) compared with the controls and the value was statistically significant (73.6% versus 55.2%, chi-square (x^2^)=5.264, p=0.022). A total of 16 (6.5%) of the WLWHA nursed their infants with breast milk substitutes (ERF) alone in the first six months of life compared to only one (0.4%) in the controls. Their feeding practices are summarized in Table 2.

**Table 2:** Infant-feeding practices among the respondents *Statistically significant

Practices	Study arm	Control arm	Chi-square test	P-value
	N=246, n (%)	N=246, n (%)		
Exclusive breastfeeding	181 (73.6)	137 (55.7)	5.264	0.022*
Mixed feeding	49 (19.9)	108 (43.9)	44.873	0.001*
Breast milk substitute only	16 (6.5)	1 (0.4)	51.655	0.001*
Early initiation of breastfeeding	173 (70.33)	86 (34.96)	63.110	0.001*
Prolonged breastfeeding (≥1 year)	212 (86.2)	210 (85.4)	0.67	0.796
Total	246 (100)	246 (100)		

The study also revealed that WLWHA were significantly more likely to initiate breastfeeding early (within one hour of childbirth) compared to the control: 173 women (70.33%) among the study arm versus 86 (34.96%) in the control group initiated breastfeeding early [p=0.001; odds ratio (OR)=3.703; 95% confidence interval (CI): 2.546 to 5.386]. A vast majority of the women in both groups breastfed their infants for up to one year. There was no significant difference between the two groups in this regard: 212 (86.2%) in the study arm and 210 (85.4%) in the control group (p=0.796; OR: 0.935; 95% CI: 0.564 to 1.552).

Determinants of exclusive breastfeeding and early initiation of breastfeeding among the respondents

In determining the factors that influenced breastfeeding practices among the 475 respondents who breastfed their infants (EBF plus mixed feeding) regardless of HIV status, the study showed that maternal age and place of residence were the main positive determinants of EBF practice among the women. Women aged 30 years and older had higher odds for the practice of EBF compared to younger women. Similarly, urban dwellers were more likely to practice EBF compared to women living in rural areas. The effects of sociodemographic factors on EBF practices and their statistical values are summarized in Table 3.

**Table 3:** Determinants of exclusive breastfeeding among the respondents 17 of the 492 respondents practiced exclusive replacement feeding (ERF) *Statistically significant CI: confidence interval; OR: odds ratio; TBA: traditional birth attendant

Determinants		Exclusive breastfeeding		P-value	OR	95% CI	
		N=475					
		Yes, n (%)	No, n (%)			Lower	Upper
Age	<30 years	135 (28.4)	112 (23.6)	0.044*	5.82	4.69	9.91
	≥30 years	158 (33.3)	70 (14.7)				
Marital status	Married	284 (59.8)	163 (34.3)	0.655	1.198	0.547	2.612
	Single	17 (3.6)	11 (2.3)				
Parity	Multiparity	126 (26.5)	85 (17.9)	0.670	1.423	0.975	2.078
	Primiparity	155 (32.6)	109 (22.9)				
Place of childbirth	Health facility	258 (54.3)	147 (30.9)	0.545	1.175	0.897	1.978
	Home/TBA	43 (9.1)	27 (5.7)				
Place of residence	Rural	156 (32.8)	123 (25.9)	0.001*	4.95	3.34	7.89
	Urban	145 (30.5)	51 (10.7)				
Income status	Good/high	189 (39.8)	137 (28.8)	0.161	1.327	0.893	1.974
	Poor/low	88 (18.5)	61 (12.8)				
Level of education	Higher education	213 (44.8)	130 (27.4)	0.568	0.885	0.581	1.348
	Lower or no education	79 (16.6)	53 (11.2)				
Availability of domestic help	Yes	199 (41.9)	147 (30.9)	0.171	1.3914	0.882	2.018
	No	77 (16.2)	52 (10.9)				
Mode of delivery	Vaginal	196 (41.3)	109 (22.9)	0.118	1.358	0.924	1.996
	Cesarean section	102 (21.5)	68 (14.3)				

In assessing for factors that favored early initiation of breastfeeding among the respondents, our analysis showed that the mode of delivery was the only obstetric factor that significantly influenced early breastfeeding positively. Women who delivered vaginally commenced breastfeeding earlier than women who delivered by cesarean section (p=0.001; OR: 3.135; 95% CI: 2.130 to 4.616). Also, urban dwellers commenced breastfeeding earlier than those in rural communities (p=0.002; OR: 5.58; 95%CI: 3.85 to 8.07). The details are shown in Table 4.

**Table 4:** Determinants of early initiation of breastfeeding 17 of the 492 respondents practiced exclusive replacement feeding (ERF) *Statistically significant CI: confidence interval; OR: odds ratio; TBA: traditional birth attendant

Determinants		Frequency		P-value	OR	95% CI	
		N=475					
		Early, n (%)	Late, n (%)			Lower	Upper
Age	<30 years	141 (29.7)	120 (25.3)	0.905	1.022	0.716	1.458
	≥30 years	113 (23.8)	101 (21.3)				
Marital status	Married	241 (50.7)	208 (43.8)	0.609	1.220	0.569	2.617
	Single	12 (2.5)	14 (2.9)				
Place of residence	Rural	138 (29.1)	142 (29.9)	0.002*	5.58	3.85	8.07
	Urban	124 (26.1)	71 (14.9)				
Income	>$300/month	176 (37.1)	148 (31.2)	0.816	1.047	0.712	1.539
	≤$300/month	82 (17.3)	69 (14.5)				
Education	Higher level of education	184 (38.7)	158 (33.3)	0.919	0.966	0.647	1.442
	Lower/no level of education	74 (15.6)	59 (12.4)				
Mode of delivery	Vaginal delivery	207 (43.6)	105 (22.1)	0.001*	3.135	2.130	4.616
	Cesarean section	55 (11.6)	108 (22.7)				
Place of delivery	Health facility	232 (48.8)	203 (42.7)	0.966	1.014	0.53	1.943
	Home/TBA	22 (4.6)	18 (3.8)				
Parity	Multiparity	117 (24.6)	87 (18.3)	0.225	1.250	0.872	1.741
	Primiparity	142 (29.9)	129 (27.1)				

Findings from the in-depth interviews among WLWHA

Findings from the in-depth discussion suggested that the main source of information and knowledge on infant feeding was government healthcare facilities, especially during the antenatal and immediate postpartum periods. Social media, friends/colleagues, and books were less common sources. The women cited that the benefits of EBF were predominantly the components of antenatal counseling and PMTCT clinics. The dangers of mixed feeding with regard to the increased risk of vertical transmission of HIV were the main reasons many of them practiced EBF. Availability, acceptability, and sustainability of the method of feeding also favored EBF. Cultural beliefs carried both positive and negative influences. Positively, cultural factors in this setting favored breastfeeding. Negatively, women who had commenced their infants on ERF from birth while in the hospital later put their infants to the breast (mixed feeding) at home within six months of birth because of pressure from family, relatives, and friends, especially when their seropositive status was not disclosed to extended family members and associates. Perception of the inadequacy of breast milk also led to practicing mixed feeding; some women felt that their babies cried excessively because they were not satisfied with breast milk alone. Early cessation of breastfeeding (<1 year) was blamed on breast health issues such as cracked nipples, mastitis, and breast abscess as well as concern about the impact on maternal health. Two women among the few who gave their infants formula feed alone (ERF) were also taking anti-tuberculosis medication and this was their main reason for not putting their infant to the breast. The extracts from the focused interview are presented in Table 5.

**Table 5:** Extracts from focused interviews

Arguments in support of exclusive breastfeeding
“I gave my infant breast milk alone in the first six months of life because of the numerous benefits they told us it entailed during the period of antenatal care”
“If I don’t breastfeed, my infant would fall sick”
“During the counseling session, the senior nurse told us that we should not give our baby breast milk and cow milk at the same time because doing so will increase HIV transmission risk”
“From our antenatal classes, we were told that breastfeeding improves baby’s health and development”
“It is a taboo in our culture not to allow your baby to suck your breast”
“Giving my infant breast milk alone was not easy. Thank God for my husband, who gave me the necessary support and encouragement”
“We were told in the hospital that when the viral load is low, the breast milk is safe for the infant”
“I learned many things about the benefits of breastfeeding from the counseling session. I also read a lot about infant feeding. I can say that exclusive breastfeeding is the best for my child”
Arguments in support of mixed feeding
“I and my husband had planned to give our baby artificial milk alone for the first six months of life. We even purchased a carton of NAN 1 but we could not continue due to pressure from our extended family members; we initiated breastfeeding when the baby was three months old”
“I commenced my baby on infant formula for the first month; however, my mother-in-law came to visit and was worried that I was not putting the baby to the breast. She even told me that it is against our culture not to breastfeed and that not suckling will make me develop breast cancer”
“If I breastfeed all my children, my breast would fall flat”
“Breast milk alone is not enough. My baby keeps crying until I add a little cow milk”
“As a working-class woman, I think exclusive breastfeeding is only possible during the maternity leave period. When I resumed work at the fourth month of delivery, it was difficult to continue with exclusive breastfeeding”
“I think if I express breast milk and keep it to be given to my child, it may get spoilt due to epileptic power supply”
Arguments in support of infant feeding
“I give my infant formula feed because of my health. I was placed on anti-tuberculosis medication”
“I was newly diagnosed and just started the anti-HIV medication”
“I did not want to take any risk at all so I gave my infant formula feed throughout his first six months. I had planned for it during the period of the pregnancy”

## Discussion

The study revealed that WLWHA practiced EBF far more than uninfected women in the region (73.6% versus 55.7%). When compared to the National Exclusive Breastfeeding rate of 29%, we found that the respondents in both groups performed better with regard to the nationally recommended infant-feeding practices [15]. The national policy recommends that children under the age of six months be exclusively breastfed [15]. However, the 55.7% prevalence of EBF among the HIV-negative women in this study was similar to the EBF prevalence of 50.5% reported from southeastern Nigeria [9]. Ours was a health facility-based research on women who were receiving care in those facilities while NDHS is usually a community-based survey. Healthcare counseling offers opportunities for behavioral change including infant-feeding options.

The prevalence of EBF of 73.6% among WLWHA in this study was higher than the EBF prevalence of 61% reported among HIV-positive mothers in the study from southwestern Nigeria [12]. In that study, much more women (26.0%) than ours (6.5%) gave their infants ERF in the first six months of life. However, the prevalence of mixed feeding in the two studies was almost similar (19.9% and 13.0%). Furthermore, a study in Abuja, the Country’s capital, among HIV-positive mothers reported a prevalence of 46%, 40%, and 14% for EBF, ERF, and mixed feeding respectively [25]. The practice of ERF depends on the socioeconomic status of the individual and, similarly, its prevalence within a locality is influenced by the prevailing social factors and income status, which may differ from one region to another even in the same country.

Nursing an HIV-exposed baby with ERF in the first six months of the infant’s life is highly effective in preventing postnatal vertical transmission of the HIV virus [26-28]. However, in low-resource settings, it is often difficult for mothers to meet the criteria for ERF. And when these conditions are not met, it is recommended that WLWHA practice EBF for the first six months of birth [29,30]. In this setting, where the national authority supports and promotes breastfeeding, mothers with HIV-exposed infants are encouraged to exclusively breastfeed their babies for the first six months of life [15]. As part of PMTCT policy, nevirapine prophylaxis is recommended to offer protection to the baby against vertical transmission of HIV when the infant is breastfed exclusively for six months. Several studies have reported near-zero transmission rates when nevirapine was given to exposed infants who received breast milk exclusively [7,21,25,31,32].

Early initiation of breastfeeding (within the first hour of birth) carries positive benefits for the mother and the newborn [33-35]. While providing the infants with timely and optimal nutrition, the risk of primary postpartum hemorrhage in the mother from uterine atony is reduced due to the effect of endogenous oxytocin on the uterus. Nipple suckling stimulates the release of oxytocin from the pituitary gland, which acts on the uterus to enhance contraction. In a systematic review, early breastfeeding was associated with reduced risk of neonatal morbidity and mortality.

Furthermore, women who initiate breastfeeding early are more likely to practice EBF for the first six months of infants’ lives and are also able to breastfeed for a longer duration [36,37]. This evidence is reinforced in this study where the WLWHA initiated breastfeeding earlier than the uninfected women (70.33% versus 34.96%) and also practiced EBF far more commonly than the controls. The proportion of the women in the control group who initiated breastfeeding early (34.96%) was similar to the pattern reported among women in the Asian continent (29-45%) [38].

In analyzing the factors that influence the respondents’ breastfeeding practices, we found that urban dwelling and maternal age of 30 years and above were significant determinants of EBF while vaginal delivery and residing in urban areas significantly increased the odds of early initiation of breastfeeding. Similar findings were reported in other studies [12,36,37]. Generally, women of advanced reproductive age dwelling in urban areas are more likely to be of higher social status, exposed to medical information, and hence in a better position to take positive decisions regarding their health and those of their infants.

Several studies have linked cesarean delivery with increased odds of delayed initiation of breastfeeding [38-40]. The duration of surgery and the anesthetic techniques and medication used as well as the time spent in the recovery room before the mothers are reunited with the infants are possible factors responsible for the delay. Also, fetal conditions in high-risk pregnancies that may necessitate operative delivery may also warrant neonatal intensive care admission immediately after delivery.

Based on the focused group interviews among the WLWHA, the study found that antenatal voluntary counseling and testing (VCT), as a component of the PMTCT program, was the main reason for the high prevalence of EBF among these women. Evidence suggests that increased utilization of ANC services has improved the impact of PMTCT in developing countries [41]. ANC offers a platform for health education for rural women who may not have access to the internet, books, and social media. It also gives an opportunity for follow-up on adherence by the skilled healthcare provider. Social and cultural factors still play a major role in infant-feeding practices among women. Some women often change their decisions in favor of the prevailing societal beliefs.

Apart from socioeconomic factors, non-disclosure of HIV status was one of the reasons women who initiated ERF could not sustain it for six months. In another study, replacement feeding was the least prevalent mode (2%) [17]. The majority who chose ERF during ANC could not sustain it beyond two months in that setting. Mothers’ education and family support were factors that favored EBF in our study. Similar factors were identified among women attending PMTCT programs in India [8]. Although family income did not significantly affect EBF in our study, financial dependence on the male partner, social stigma, and cultural beliefs about infant nutrition have been cited as major factors hindering postnatal PMTCT programs [8].

A significant proportion of the women breastfed their infants for more than one year. Prolonged breastfeeding of one year and beyond was promoted by the knowledge of its benefits to infants’ health and wellbeing. Reduced incidences of infants’ illnesses and hospitalization due to diarrheal diseases and respiratory infections as well as the perception of a reduction in the incidence of mothers’ malignant breast diseases later in life strongly favored longer duration of breastfeeding among WLWHA. The World Health Organization (WHO) recommends EBF for six months, then the addition of complementary feed after six months, and continued breastfeeding for two years [10].

While socioeconomic and cultural factors were responsible for mixed feeding, concerns about maternal comorbidities such as pulmonary tuberculosis and perceived high viral loads in newly diagnosed individuals were medical reasons for practicing ERF by some of the women. In a randomized trial in Botswana [27], ERF was more effective than breastfeeding with zidovudine prophylaxis in preventing postnatal HIV vertical transmission but breastfeeding was associated with a lower infant mortality rate at seven months compared to ERF. However, HIV-free survival at 18 months was comparable in both arms. When the criteria for ERF are not met, HIV-positive clients with newborn babies should be encouraged to adhere to ARV medication throughout the period of breastfeeding and their infants should be given ARV prophylaxis according to WHO recommendations [41].

Strengths and limitations of the study

The mixed nature of the study enabled us to obtain information that might not have been captured on the quantitative data questionnaire. The main limitation was relying on information obtained from the respondents. It is possible that important information may not have been volunteered. However, this was reduced to the minimum by reassuring the respondents of confidentiality.

## Conclusions

The high prevalence of EBF among WLWHA compared to the controls in this study is a reflection of the achievement of the PMTCT programs in mitigating the effects of the HIV pandemic in sub-Saharan African countries. Lessons learned from this program may be adopted in improving breastfeeding practices in the general population and promoting infants' survival through nutritional counseling during ANC. However, the low prevalence of ERF due to cultural influences may be associated with high levels of mixed feeding in some regions. Hence, other PMTCT programs, especially with regard to ARV medications, should be promoted.

## References

[1] NEOVITA Study Group (2016). Timing of initiation, patterns of breastfeeding, and infant survival: prospective analysis of pooled data from three randomised trials. Lancet Glob Health.

[2] Iacovou M, Sevilla A (2013). Infant feeding: the effects of scheduled vs. on-demand feeding on mothers' wellbeing and children's cognitive development. Eur J Public Health.

[3] (2023). UNAIDS and WHO: report on the global AIDS epidemic. https://www.unaids.org/globalreport.

[4] Maredza M, Bertram MY, Saloojee H, Chersich MF, Tollman SM, Hofman KJ (2013). Cost-effectiveness analysis of infant feeding strategies to prevent mother-to-child transmission of HIV in South Africa. Afr J AIDS Res.

[5] (2019). UNICEF: UN updated framework for priority action on HIV and infant feeding. https://data.unaids.org/publications/external-documents/who_infantfeedingframework_en.pdf.

[6] Yu W, Li C, Fu X (2014). The cost-effectiveness of different feeding patterns combined with prompt treatments for preventing mother-to-child HIV transmission in South Africa: estimates from simulation modeling. PLoS One.

[7] (2023). World Health Organization: consolidated guidelines on the use of antiretroviral drugs for treating and preventing HIV infection. https://www.who.int/publications/i/item/9789241549684.

[8] Suryavanshi N, Mave V, Kadam A (2018). Challenges and opportunities for outreach workers in the Prevention of Mother to Child Transmission of HIV (PMTCT) program in India. PLoS One.

[9] Obiora OL, Ezenduka PO, Umeonwuka C (2019). Infant feeding practices among parturient women in rural communities of Anambra State, Nigeria. Int J Community Med Public Health.

[10] Naja F, Chatila A, Ayoub JJ, Abbas N, Mahmoud A, Abdulmalik MA, Nasreddine L (2022). Prenatal breastfeeding knowledge, attitude and intention, and their associations with feeding practices during the first six months of life: a cohort study in Lebanon and Qatar. Int Breastfeed J.

[11] Chantry CJ, Howard CR, Auinger P (2006). Full breastfeeding duration and associated decrease in respiratory tract infection in US children. Pediatrics.

[12] Aishat U, David D, Olufunmilayo F (2015). Exclusive breastfeeding and HIV/AIDS: a crossectional survey of mothers attending prevention of mother-to-child transmission of HIV clinics in southwestern Nigeria. Pan Afr Med J.

[13] Smith MM, Kuhn L (2000). Exclusive breast-feeding: does it have the potential to reduce breast-feeding transmission of HIV-1?. Nutr Rev.

[14] (2019). National Population Commission (NPC) [Nigeria] and ICF International 2014. Nigeria Demographic and Health Survey. Abuja, Nigeria and Rockville, Maryland, USA: NPC and ICF International. https://dhsprogram.com/pubs/pdf/fr293/fr293.pdf.

[15] (2019). Nigeria Demographic and Health Survey 2018. National Population Commission, Abuja, Nigeria Feeding Infant and Young Children Practices, Breastfeeding Status by Age. https://www.dhsprogram.com/pubs/pdf/FR359/FR359.pdf.

[16] Ogundele T, Ogundele OA, Adegoke AI (2019). Determinants of prelacteal feeding practices among mothers of children aged less than 24 months in Ile-Ife Southwest Nigeria: a community cross-sectional study. Pan Afr Med J.

[17] Kinyua JG, Lihana RW, Kiptoo MK, Mwangi J, Okoth V, Onyango R, Andang’o P (2016). Factors associated with choice of infant feeding practices among HIV-1 positive post-natal clinic attendees in Tharaka Nithi county. J Health Med Nurs.

[18] Mattar L, Hobeika M, Zeidan RK, Salameh P, Issa C (2019). Determinants of exclusive and mixed breastfeeding durations and risk of recurrent illnesses in toddlers attending day care programs across Lebanon. J Pediatr Nurs.

[19] Desmond C, Bland RM, Boyce G, Coovadia HM, Coutsoudis A, Rollins N, Newell ML (2008). Scaling-up exclusive breastfeeding support programmes: the example of KwaZulu-Natal. PLoS One.

[20] Nabwera HM, Jepkosgei J, Muraya KW (2017). What influences feeding decisions for HIV-exposed infants in rural Kenya?. Int Breastfeed J.

[21] (2019). World Health Organization. Guidelines on HIV and infant feeding 2010: principles and recommendations for infant feeding in the context of HIV and a summary of evidence. https://apps.who.int/iris/handle/10665/44345.

[22] Wise J (2001). Breast feeding safer than mixed feeding for babies of mothers with HIV. BMJ.

[23] Maru S, Datong P, Selleng D (2009). Social determinants of mixed feeding behavior among HIV-infected mothers in Jos, Nigeria. AIDS Care.

[24] Kobby B (2020). Nigeria National HIV prevalence drops to 1.4 percent. Abuja-Geneva. https://www.unaids.org/en/resources/presscentre/pressreleaseandstatementarchive/2019/march/20190314_nigeria.

[25] Mohammed A, Shehu AU, Zoaka AI (2010). Infant feeding options, practices and determinants of feeding practices among HIV seropositive mothers in Abuja, Nigeria. Niger Med J.

[26] Wyckoff AS (2013). HIV-infected mothers should not breastfeed, even if taking antiretroviral drugs: policy. AAP.

[27] Coovadia HM, Brown ER, Fowler MG (2012). Efficacy and safety of an extended nevirapine regimen in infant children of breastfeeding mothers with HIV-1 infection for prevention of postnatal HIV-1 transmission (HPTN 046): a randomised, double-blind, placebo-controlled trial. Lancet.

[28] Thior I, Lockman S, Smeaton LM (2006). Breastfeeding plus infant zidovudine prophylaxis for 6 months vs formula feeding plus infant zidovudine for 1 month to reduce mother-to-child HIV transmission in Botswana: a randomized trial: the Mashi Study. JAMA.

[29] (2019). HIV initiative 'prevention of mother-to-child transmission; to saves exposed infants in Nigeria. https://www.afro.who.int/news/hiv-initiative-prevention-mother-child-transmission-saves-exposed-infants-nigeria.

[30] (2023). National Agency for the Control of AIDS (NACA). Factsheets: national situation and needs assessment of HIV/AIDS, drugs use and related health services in Nigerian prisons. https://www.unodc.org/documents/nigeria//HIV_Prisons_Full_Study_Report_.pdf.

[31] Oguta T, Omwega A and Sehmi J (2023). Oguta T, Omwega A, Sehmi J: infant feeding alternatives for HIV positive mothers in Kenya. Field exchange.

[32] Potty RS, Sinha A, Sethumadhavan R, Isac S, Washington R (2019). Incidence, prevalence and associated factors of mother-to-child transmission of HIV, among children exposed to maternal HIV, in Belgaum district, Karnataka, India. BMC Public Health.

[33] Takahashi K, Ganchimeg T, Ota E (2017). Prevalence of early initiation of breastfeeding and determinants of delayed initiation of breastfeeding: secondary analysis of the WHO Global Survey. Sci Rep.

[34] Cox KN, Giglia RC, Binns CW (2015). The influence of infant feeding attitudes on breastfeeding duration: evidence from a cohort study in rural Western Australia. Int Breastfeed J.

[35] Beijers R, Cillessen L, Zijlmans MA (2016). An experimental study on mother-infant skin-to-skin contact in full-terms. Infant Behav Dev.

[36] Wu Y, Wang Y, Huang J (2018). The association between caesarean delivery and the initiation and duration of breastfeeding: a prospective cohort study in China. Eur J Clin Nutr.

[37] Liben ML, Yesuf EM (2016). Determinants of early initiation of breastfeeding in Amibara district, Northeastern Ethiopia: a community based cross-sectional study. Int Breastfeed J.

[38] Sharma IK, Byrne A (2016). Early initiation of breastfeeding: a systematic literature review of factors and barriers in South Asia. Int Breastfeed J.

[39] Khan J, Vesel L, Bahl R, Martines JC (2015). Timing of breastfeeding initiation and exclusivity of breastfeeding during the first month of life: effects on neonatal mortality and morbidity--a systematic review and meta-analysis. Matern Child Health J.

[40] Prior E, Santhakumaran S, Gale C, Philipps LH, Modi N, Hyde MJ (2012). Breastfeeding after cesarean delivery: a systematic review and meta-analysis of world literature. Am J Clin Nutr.

[41] Mutabazi JC, Zarowsky C, Trottier H (2017). The impact of programs for prevention of mother-to-child transmission of HIV on health care services and systems in sub-Saharan Africa - a review. Public Health Rev.

